# Protocol for a hybrid type 3 cluster randomized trial of a technical assistance system supporting coalitions and evidence-based drug prevention programs

**DOI:** 10.1186/s13012-021-01133-z

**Published:** 2021-06-25

**Authors:** Louis D. Brown, Sarah M. Chilenski, Rebecca Wells, Eric C. Jones, Janet A. Welsh, Jochebed G. Gayles, Maria E. Fernandez, Damon E. Jones, Kimberly A. Mallett, Mark E. Feinberg

**Affiliations:** 1 School of Public Health in El Paso, The University of Texas Health Science Center at Houston, 5130 Gateway East Blvd., Rm 316, El Paso, TX 79905 USA; 2 Edna Bennett Pierce Prevention Research Center, The Pennsylvania State University, State College, USA; 3 School of Public Health, The University of Texas Health Science Center at Houston, Houston, USA

**Keywords:** Community coalitions, Technical assistance, Interactive Systems Framework, Risk reduction behavior, Substance-related disorders, Prevention, Adolescent behavior, Implementation support, Sustainability

## Abstract

**Background:**

Over 5000 community anti-drug coalitions operating in the USA serve as a cornerstone of federal drug prevention. These coalitions, however, have demonstrated effectiveness in preventing substance use only when they use technical assistance (TA) and implement evidence-based programs (EBPs). The absence of TA and EBP implementation by coalitions is a key research-to-practice gap. The Coalition Check-Up TA system is designed to fill this gap by supporting community coalition implementation of EBPs. Existing TA models for evidence-based coalition approaches are resource intensive and coalition model specific. The Coalition Check-Up is a lower cost strategy that works with a variety of types of coalitions to support sustainable implementation of EBPs. This study protocol describes a hybrid type 3 effectiveness-implementation trial applying Wandersman’s Interactive Systems Framework to test the effects of the Coalition Check-Up on coalition EBP implementation capacity and outcomes. The Interactive Systems Framework outlines how the prevention support system—especially TA—bolsters EBP dissemination and implementation.

**Methods:**

Using a cluster randomized controlled design, this trial will test the overall effectiveness of the Coalition Check-Up, including how it contributes to EBP implementation and prevention of youth substance use. The first aim is to estimate the impact of the Coalition Check-Up on coalitions’ capacity to do their work. We will recruit 68 anti-drug coalitions for random assignment to the Coalition Check-Up or “TA as usual” condition. We will evaluate whether the Coalition Check-Up improves coalition capacity using measures of coalition member responses about team processes, coalition network composition, and collaborative structure. Our second aim is to estimate the impact of the Coalition Check-Up on implementation of EBPs, and our third aim is to estimate the impact of the Coalition Check-Up on youth substance use.

**Discussion:**

This project will clarify how the Coalition Check-Up, a scalable approach to TA due to its low cost, affects coalition capacity to support EBP implementation. Analyses also provide insight into causal pathways from the prevention support system to the prevention delivery system outlined by the Interactive Systems Framework. Results will build the evidence-base for how to support community coalitions’ sustainable implementation of evidence-based prevention programs and policies.

**Trial registration:**

Clinicaltrials.gov registration number NCT04592120. Registered on October 19, 2020.

Contributions to the literature

- Community coalitions’ impact on public health hinges on their use of and sustainment of evidence-based programs. Yet little research investigates how to support such implementation.
- The Coalition Check-Up offers affordable technical assistance for coalitions’ use of evidence-based programs.
- This hybrid type 3 effectiveness-implementation trial tests Coalition Check-Up effects on evidence-based program implementation for drug prevention.
- The study is one of the first to test causal pathways outlined by the Interactive Systems Framework for Dissemination and Implementation.
- This study tests an innovation in the audit and feedback process—using motivational interviewing to support action planning based on feedback.

## Background

Several federal entities fund more than 5000 community anti-drug coalitions in the USA [1–3]. Such coalitions are attractive because they support community-driven solutions to local problems [4]. Community coalitions also promote processes that improve program implementation quality, including interorganizational collaboration, shared decision-making, and communication [5]. They can also support evidence-based practices (EBP) by serving as hubs of prevention expertise and support for the program delivery system—coordinating EBP selection, training, monitoring, evaluation, and problem-solving [5, 6]. For these reasons, coalitions provide potential key mechanisms for bridging the research-to-practice gap in substance use prevention.

### Technical assistance for community coalitions

Currently, there is insufficient knowledge about how to support coalitions’ EBP implementation. Community coalition models have demonstrated effectiveness only when they have entailed comprehensive TA and EBP implementation [7–10]. TA is particularly important for EBP implementation because EBPs require technical expertise, coaching, coordination among partners, and monitoring [11–13]. However, such TA often costs more than coalitions can afford. For instance, PROSPER and Communities That Care, two coalition-based approaches to prevention, have demonstrated effectiveness in implementing EBPs. Both entailed a 25% Full-Time-Equivalent TA provider per coalition [14, 15]. Budget-constrained TA providers often use group-based regional trainings and webinars that are not individualized or evidence-based [16]. Even individualized TA typically does not determine if coalitions are self-diagnosing correctly or addressing relevant capacities [17, 18]. Coalitions struggling to implement EBPs are often disconnected and do not reach out for help [19]. Currently, there is no evidence-based model for low or moderately intensive, sustainable TA.

As a TA model, the Coalition Check-Up requires 84% less TA time compared to the TA used in Communities That Care and PROSPER (4% FTE per coalition vs. 25% FTE). The Coalition Check-Up TA model is a low-intensity yet tailored solution to the tension between TA effectiveness and cost constraints. It is not specific to a particular community coalition model (e.g., PROSPER) or EBP, instead focusing on common dimensions of coalition capacity and program implementation intended to improve youth outcomes [20, 21]. Such a “light touch” intervention would be more easily sustained and is more likely to be adopted to support sustainability on a national scale than existing evidence-based TA models. Additionally, we do not yet know how coalitions can sustain implementation once TA diminishes. Although coalitions may continue to operate, their engagement and activities tend to decline when TA funding ends [22]. After TA loss, some coalitions have seen large declines in recruitment rates and implementation quality for prevention programming [22, 23]. One potential benefit of the Coalition Check-Up is preventing the usual loss of functioning among coalitions as they lose funding for TA. If effective, the Coalition Check-Up will provide lower-cost and thus more sustainable TA to help community coalitions implement EBPs.

### Coalition Check-Up conceptual framework

**Fig. 1:**
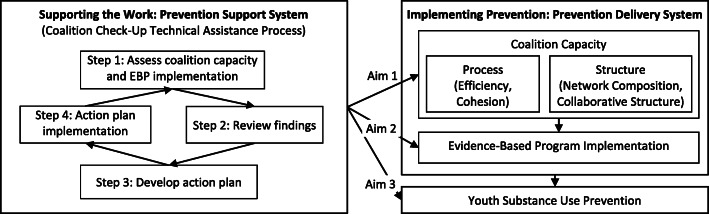
Coalition Check-Up Conceptual Framework (adapted from Interactive Systems Framework)

To help bridge these research-to-practice gaps, this study design applies the Interactive Systems Framework for Dissemination and Implementation. This framework focuses on how the prevention support system—especially TA—bolsters EBP dissemination and implementation. Figure 1 depicts how TA providers work with practitioners implementing EBPs in the prevention delivery system. Coalition Check-Up TA supports coalition capacity and EBP implementation, which are expected to subsequently contribute to reductions in youth substance use. Figure 1 illustrates aims 1–3 for testing these hypothesized paths of influence.

Previous studies suggest that the coalition capacities shown in Fig. 1 are necessary to support EBP implementation [14, 21, 22, 24, 25]. Internal team processes such as group cohesion foster trust needed for sustained EBP implementation [22, 26–29]. Coalition network structure also influences coalition outcomes [25, 30]. For example, coalitions whose members seek advice outside their area of expertise have more support from community leaders and organizations, plus more detailed sustainability plans [30].

In Fig. 1, the 4-step Coalition Check-Up TA process provides proactive data-driven continuous quality improvement cycles that TA research suggests are particularly useful [17]. Step 1 in the Coalition Check-Up is assessing critical dimensions of the coalition’s capacity and EBP implementation. A coalition profile based on assessment data is reviewed in Coalition Check-Up step 2. Here the TA provider works with the coalition to consider several dimensions of coalition capacity and EBP implementation while celebrating strengths and prioritizing areas for growth. Once areas for growth are prioritized, the TA provider uses structured action planning in step 3 to help coalition members establish consensus on how to improve each. For example, the Coalition Check-Up may identify network structural characteristics such as centralization of influence and information as problematic [31]. The TA provider can then help a coalition develop an action plan to share decision-making power more broadly. To support step 3, TA providers draw upon a user-friendly guidebook, based on a comprehensive review of research for improving each dimension of coalition capacity and EBP implementation in the Coalition Check-Up assessment. This guidebook replaces otherwise improvised planning efforts and can speed decision making, thus reducing TA and coalition burden in creating action plans. In step 4, TA providers review and support progress on action plan implementation with the coalition. Efforts are evaluated every year after the initial assessment in a continuous quality improvement cycle.

Central to the Coalition Check-Up is its use of an “audit and feedback” implementation strategy, whereby performance data is collected and reviewed to inform continued improvement [32]. Following recommendations from the audit and feedback literature, the Coalition Check-Up identifies and addresses coalition and EBP implementation capacity deficiencies that frequently lead to failure [33]. A key innovation of the Coalition Check-Up is its use of motivational interviewing to support action planning and implementation based on an audit and feedback. TA providers use motivational interviewing techniques to cultivate a coalition-driven change process. By clarifying the benefits of change and addressing barriers without imposing recommendations, TA providers can guide coalitions to embrace change. These processes strengthen coalition motivation to develop and execute their own action plans [34].

We have found that without the structured Coalition Check-Up action planning process, coalitions do not typically use assessment reports or ask for help in doing so. For example, when a technical assistance provider faced budget cuts, they made face-to-face meetings to discuss evaluation data available only on request; only a small percentage (approximately 15%) of coalitions pursued them [35]. Thus, the Coalition Check-Up assessment with TA process appears to be critical for proactively promoting both motivation and capacity to use the data to make decisions about next steps, thereby improving EBP implementation quality [36].

Limited use of feedback for improvement purposes is a key challenge for audit and feedback as an implementation strategy [33]. Evidence suggests that audit and feedback interventions yield only modest improvements in practice [37]. To test the potential of more proactive TA to actualize benefits of audit and feedback, the current study compares coalitions receiving a rudimentary audit and feedback process (“TA as usual”), to those receiving the same audit and feedback materials combined with brief TA outreach that employs motivational interviewing to support action planning and action plan implementation. Specifically, we test the moderating influence of two core processes thought to strengthen audit and feedback: (1) fidelity to motivational interviewing techniques and (2) the collaborative working relationship between the person providing feedback and the coalition. We also test two factors thought to mediate the influence of audit and feedback on outcomes: (1) action plan quality and (2) action plan implementation [38].

### Objectives and aims

The testing of audit and feedback processes occurs as part of our larger main objective, which is to test the overall effectiveness of the Coalition Check-Up, including how it contributes to EBP implementation and prevention of youth substance use. Building on the Interactive System Framework, our central hypothesis is that the Coalition Check-Up enhances communities’ prevention support systems, thereby increasing coalition capacity for EBP implementation and the probability that EBPs will reduce youth substance use. This research will build the evidence-base for a scalable TA model, indicating how to intervene with community coalitions to maximize EBP fidelity and sustainability. To achieve our objectives, we will test this central hypothesis by pursuing three specific aims:

#### Aim 1

Estimate the impact of the Coalition Check-Up on coalition capacity. Coalitions will be randomly assigned to the Coalition Check-Up or “TA as usual” condition. We will evaluate whether the Coalition Check-Up improves coalition capacity using measures based on coalition member reports of team processes, network composition, and collaborative structure.

#### Aim 2

Estimate the impact of the Coalition Check-Up on implementation of evidence-based programs. We will test the hypothesis that coalitions receiving the Coalition Check-Up will implement EBPs with greater: (a) quantity, (b) quality, and (c) sustainability. We will also test coalition capacity as a mediator of Coalition Check-Up impact on EBP implementation.

#### Aim 3

Estimate the impact of the Coalition Check-Up on youth substance use. We will test the hypothesis that communities participating in the Coalition Check-Up will reduce youth substance use, including alcohol, tobacco, marijuana, and opioids, relative to communities whose coalitions receive TA as usual. We will also test EBP implementation as a mediator of the Coalition Check-Up impact on youth substance use.

## Method

**Fig. 2:**
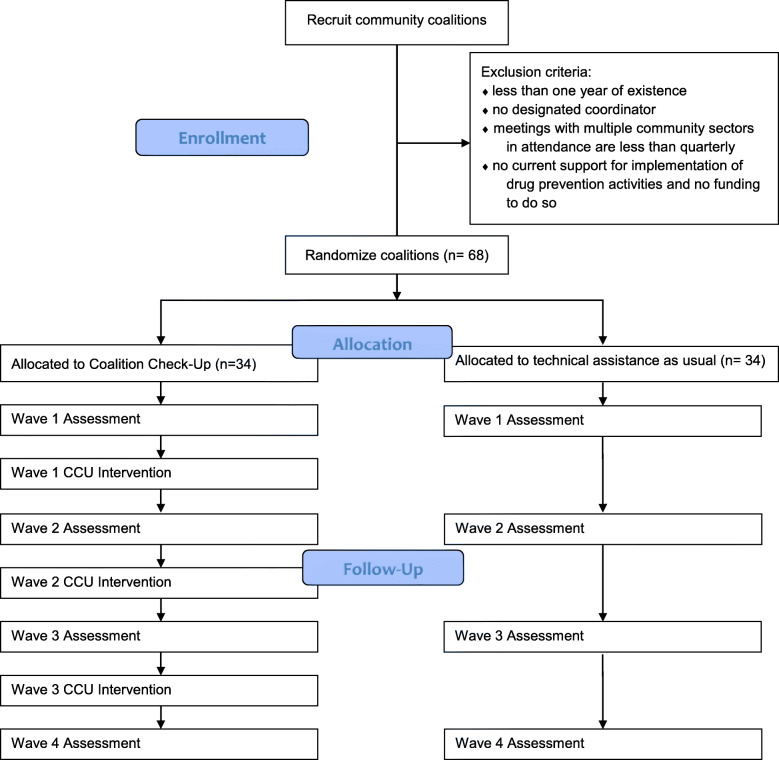
CONSORT diagram

This study is classified as a hybrid type 3 effectiveness-implementation trial, given its focus on testing a strategy for supporting EBP implementation, while still examining youth outcomes [39]. In this parallel groups cluster randomized trial, we will randomly assign existing Pennsylvania and Missouri anti-drug community coalitions to either the Coalition Check-Up or a comparison condition. Both study arms will have access to TA as usual, including regional trainings and TA by phone. As illustrated in the Fig. 2 CONSORT diagram, all community coalitions in the study will participate in the same annual assessment process and receive a report that summarizes the data collected. However, only coalitions assigned to the Coalition Check-Up will receive personalized outreach by TA providers. This creates a robust comparison because of the prior evidence that coalitions are unlikely to make effective use of the feedback reports without personalized TA.

### Coalition recruitment

We will recruit 68 coalitions from community anti-drug coalitions across Pennsylvania and Missouri, USA. This coalition population is similar to other US coalitions, which enhances study generalizability. These coalitions have received funding from a wide variety of sources, and their activity levels have fluctuated with changes in funding levels over time. Coalitions function in rural, suburban, and urban areas, ranging in age from 1 to 30 years old. We connect with these coalitions through the Evidence-based Prevention and Intervention Support (EPIS) group in the Pennsylvania State University’s Prevention Research Center. The EPIS team interfaces with community agencies, schools, and coalitions to provide support to those attempting to integrate prevention research into their practices.

We are using a layered targeted strategy to recruit coalitions, first with email communications from EPIS TA staff. We then introduce the project at coalition regional meetings organized by EPIS, providing time for questions and small group discussion to connect project researchers with coalition members. We then conduct follow-up phone calls and online meetings. The decision to participate rests on each coalition’s leadership team or at times with the full coalition.

#### Inclusion criteria

To be eligible, coalitions must (1) have been in existence for at least 1 year, (2) have a designated coordinator, (3) have at least quarterly meetings in which multiple sectors of the community attend, (4) currently support implementation of drug prevention activities or secured funding to do so, (5) be willing to complete coalition capacity and EBP implementation assessments annually, and (6) be willing to participate in four in-person meetings annually with the Coalition Check-Up TA provider.

### Randomization

Matched pairs of coalitions will be randomly assigned to the Coalition Check-Up condition or TA as usual. Coalitions will be matched on level of TA availability at baseline (0 = none, 1 = occasional trainings and group TA, 2 = regularly scheduled group TA, 3 = group TA and regularly scheduled one-on-one supervision, 4 = regularly scheduled one-on-one capacity-building TA), years in operation, coalition model employed, population density, US Census poverty rate, and availability of funding. A random number seed will be generated by an external third party using random.org. SAS Proc Surveyselect uses this random number seed to select one of each pair of coalitions recruited into the study as the intervention condition. Data collectors and data analysts will be blind to study condition, with no circumstances under which unblinding is permissible.

### Intervention procedures

The Coalition Check-Up cycle will be repeated annually, a total of three times over 3 years. Consistent with previous coalition research suggesting it takes 2–3 years to detect an effect on youth substance use, we expect three Coalition Check-Up iterations will enhance coalition capacity and EBP implementation sufficiently to detect an effect on youth substance use [40, 41].

#### Coalition Check-Up Step 1: Assess coalition capacity and EBP implementation

All coalition members will be invited to complete an assessment of coalition capacity, and program coordinators will be asked to complete an assessment of EBP implementation. A researcher then uses these data to produce accessible feedback reports that visually display findings and support coalition-driven decision-making regarding what to improve.

#### Coalition Check-Up Step 2: Review findings

In this step, the TA provider jointly reviews the coalition profile with coalition leaders and members. The TA provider facilitates the discussion, presenting the results and specific questions for reflection at key points to encourage understanding and critical thinking while celebrating strengths and identifying areas for growth.

#### Coalition Check-Up Step 3: Develop action plan

Approximately 1 month after reviewing findings in step 2, the TA provider meets with the coalition again. After a brief review of the previously identified strengths and areas for growth, TA providers can facilitate large-group discussion on the top one, two, or three priorities, or divide the coalition into smaller groups, asking each to focus on an identified area for growth. Within each discussion, participants specify an improvement goal, use the Coalition Check-Up guidebook to brainstorm and select activities to reach their goal, and delegate members and committees to lead the work. At discussion’s end, each coalition officially adopts the action plan. The coalition leaders distribute the action plan with the meeting notes.

#### Coalition Check-Up Step 4: Action plan implementation

Once the action plan notes are distributed, action plan implementation monitoring and support begin. Coalition leaders check-in with members on progress, with updates at each coalition meeting. TA providers schedule two in-person visits at coalition meetings over the next 6 months to provide assistance, suggestions, resources, and feedback. Between in-person visits, the TA provider will check in with coalition leaders at least monthly. These regular check-ins are typically short (e.g., 30–60 min) but allow for continued relationship building using motivational interviewing techniques, information sharing, problem-solving, and other prompt support [42]. Our previous research suggests monthly TA provider contact enhances coalition program implementation and sustainability [15].

### Intervention implementation monitoring

Dimensions of Coalition Check-Up implementation quality include (1) coalition-TA provider collaborative working relationship, (2) action plan quality and implementation, and (3) fidelity to the Coalition Check-Up model. The collaborative working relationship between the TA provider and coalition leader/coalition will be assessed with measures drawn from Chilenski, Feinberg, and Welsh’s prior work on PROSPER [13, 43, 44]. The scale captures trust between the TA provider and the coalition, empowerment vs. providing information-only, and mutual respect. Collaborative working relationship will be assessed quarterly by TA providers and rated annually via the coalition leader interview. Developing a collaborative working relationship is a foundational principle in motivational interviewing.

Ratings of action plan quality and implementation will employ measures developed previously by the research team [45, 46]. As in prior studies, investigators will create a scoring rubric and test it on existing coalition implementation plans external to the randomized trial. Then inter-rater reliability will be calculated, with a minimum of 30% of the implementation plans independently rated by two members of the research team [47].

Coalition Check-Up fidelity will be assessed with three measures: (1) the TA Record completed by TA providers after monthly coalition contacts, (2) the coalition leader interview, and (3) expert ratings of motivational interviewing fidelity. Through the TA Record, we will know whether the TA provider is contacting coalitions monthly and spending adequate amounts of time on each Coalition Check-Up step. The coalition coordinator interview provides a more objective perspective on Coalition Check-Up fidelity, assessing the extent to which coalitions complete each Coalition Check-Up step. Assessment of TA provider motivational interviewing fidelity relies on motivational interviewing experts coding recordings of TA sessions, noting desired and undesired motivational interviewing behaviors using Motivational Interviewing Treatment Integrity codes [48, 49]. All TA sessions will be recorded and 10% will be coded. A motivational interviewing expert will use the codes to give TA providers feedback and coaching as needed to ensure their work remains consistent with motivational interviewing principles [50].

### Data collection

#### Aim 1: Data collection—coalition capacity

Annually for 4 years, each member of a participating coalition will be invited to participate in a 20-min web-based coalition member survey, and each coalition coordinator will be asked to report on coalition operations and complete an implementation monitoring tool for community collaborations, modeled after the PROSPER and Communities That Care Milestones & Benchmarks tools. After working with the coalitions and their TA providers to obtain a coalition membership roster, the coalition leader notifies coalition members via email, announcements at meetings, and phone calls, about the survey and to convey support for participation. After the coalition leader notifies coalition members about the survey, data collection staff send the first survey invitation through email (or US mail if individuals do not use email). All respondents in intervention and control conditions receive $10 for completing each survey and their coalition receives a de-identified feedback report for strategic planning. Respondents receive up to four reminders to complete the coalition functioning survey, one per week. Finally, we call participants who have not completed the survey to troubleshoot any problems they may be having and encourage their participation.

#### Aim 2: Data collection—EBP implementation

We will annually interview coalition leaders on the coalition’s role in coordinating and sponsoring EBPs, and EBP coordinators on the EBP implementation measures—each receives $20 compensation for each interview [51]. To minimize bias, our measures of EBP implementation rely on factual information about program implementation asked in a standardized fashion across programs—such as the involvement of certified trainers, fidelity monitoring, and program adherence.

#### Aim 3: Data collection—youth substance use

We will measure youth substance use and related behaviors with the Pennsylvania Youth Survey and the Missouri Student Survey—statewide surveys similar to the Communities That Care Youth Survey [52]. In 2019, 413 out of 500 (83%) school districts in Pennsylvania participated, and in 2018, 102 of 115 (89%) counties participated in Missouri [53, 54]. The surveys are conducted free of charge in all schools in Pennsylvania and Missouri by their state governments. Survey data are collected every other year in 6th, 8th, 10th, and 12th grades. All instruments are available from the first author.

### Data management and monitoring

We will use RedCap, a secure platform for online surveys and telephone interview data entry. RedCap minimizes data entry errors because survey respondents and interviewers use the interface to directly enter data. All data collection protocols include a form on which research staff members record any problems with the data collection or adverse events. Once data entry is complete, the data is exported to SAS, where all data manipulations are automatically documented in the process of writing SAS code.

### Measures

#### Aim 1: Measures—coalition capacity

This construct is assessed annually and divided into internal team processes and coalition structural characteristics. Internal team processes include cohesion and efficiency. Cohesion (3 items, α = 0.78) measures the extent to which coalition members perceived feelings of unity, group spirit, trust, and belonging within their coalitions. Efficiency (3 items, α = .91) quantifies the work ethic, efficiency, and task focus of the coalition members.

Coalition structural characteristics consist of network composition and collaborative structure. Measures of collaborative structure under investigation are decentralization and multiplexity. Decentralization is a combination of hierarchy, connectedness, average path length, and clustering [55]. Multiplexity is the number of unique types of cooperation or interaction each partner reported with each other partner, among sharing information, personnel, monetary resources, and other cooperation [56]. Network composition measures are sectoral diversity and intersectoral communication. Following our previous research, sectoral diversity is measured using an entropy index whose value increases as the number of sectors increases and the distribution of the coalition’s membership across sectors equalizes [30]. To measure intersectoral communication, respondents name individuals in the coalition to whom they went to for advice about coalition matters. When an individual cites someone from a different sector as a source of advice, that dyad is counted as an intersectoral tie. A coalition’s intersectoral communication is measured as the coalition’s mean intersectoral ties per respondent [30].

#### Aim 2: Measures—EBP implementation

EBP implementation consists of three core facets assessed annually: (a) quantity. (b) quality, and (c) sustainability. EBP quantity is a coalition-level variable computed as the number of youth reached annually by all coalition-supported EBPs. To estimate the total number of youth reached by a particular EBP, we ask the coordinator of the EBP to report the unduplicated number of youth reached annually at each location where the program is being implemented. Thus, EBP quantity represents the sum “reach” achieved by complementary programs implemented by a coalition to address different developmental stages across the prevention spectrum.

EBP quality is a coordinator-reported program-level variable computed as a composite of 7 scales: (a) staff training (6 items, α = .91); (b) staff motivation and competence (5 items, α = .93); (c) fidelity monitoring (11 items, α = .76); (d) evaluation (13 items, α = .81); (e) dosage (2 items); (f) adherence (7 items); and (g) implementation barriers (11 items, α = .81) (21, 26).

EBP sustainability consists of two measures: (1) overall EBP sustainability and (2) sustainability planning. Overall EBP sustainability is a coalition-level variable that is the sum number of years all EBPs are in operation during years 2–4 of the project, including both existing and new EBPs. An EBP will be designated as non-operational when (a) it has no reach or (b) there is an absence of ongoing training or TA in the past 12 months and no funding available to support the program. Sustainability planning is a coordinator-reported program level outcome (12 items, α =.85) [57].

#### Aim 3: Measures—youth substance use

We use measures from the Pennsylvania Youth Survey and Missouri Youth Survey which include items measuring substance use, along with several risk and protective factors [24, 58]. There are 8 key variables under investigation in this study: dichotomized lifetime use and 30-day use of alcohol, tobacco, marijuana, and opioids, respectively [58, 59].

### Power analysis

We used Optimal Design to estimate power for cluster-randomized trials [60]. In this study, youth-level substance use data and program-level implementation data are clustered within coalitions, which is the level of random assignment. Overall, we will have a power of .80 to detect Cohen’s *d* effect size ranges as follows: aim 1, .24 to .37, a small to medium effect, depending on the measure of coalition capacity; aim 2, .31 to .42, a medium effect, depending on the EBP implementation measure; and aim 3, .25, a small effect, for measures of youth substance use.

### Data analytic plan

For aim 1, multi-level longitudinal growth models will be used to test our hypotheses that coalitions assigned to the Coalition Check-Up condition will show improving coalition capacity over time compared to the comparison condition. Our first step entails testing for baseline differences on all measures of coalition capacity between the two experimental conditions. All models will be estimated in MPlus using full information maximum likehood, which has the advantage of being able to estimate missing data [61].

We will model coalition structural characteristics (decentralization, multiplexity, sectoral diversity, intersectoral communication) as coalition-level dependent variables for aim 1. These dependent variables will be predicted by the experimental condition (0=control; 1= Coalition Check-Up), controlling for potential confounders (e.g., coalition model). Once we have multiple waves of data, measurement occasion will be repeated within coalition, and we will utilize growth modeling in our tests of Coalition Check-Up impact on coalition capacity. We will include pretest (i.e., year 0) measures in the model, enabling us to examine change in these characteristics over time between the two groups. Analyses will start by testing unconditional longitudinal models with maximum likelihood estimation in order to find the best functional form, after which we will test our hypotheses [62]. We will identify the trajectory of coalition capacity using model fit indices—including the AIC, BIC, and the −2 Log Likelihood deviance across different types of longitudinal models, including (a) the empty model (i.e., model with only a random intercept), (b) polynomial growth models, (c) polynomial growth models with random slopes, and (d) piecewise models [13, 62]. Once the developmental trajectory of coalition capacity is properly modeled, it will be predicted by experimental condition (0=control; 1= Coalition Check-Up), controlling for potential confounders.

Member experiences of coalition cohesion and efficiency will be modeled at the member level. Coalition member reports here are nested within coalitions. We will estimate a random intercept for the internal team processes in each analytic model. The effect of the Coalition Check-Up is entered at the level of coalition. We will start by testing for baseline differences in coalition capacity between the two conditions. The longitudinal data will add a third level to this model, necessitating use of growth modeling. We will use the same process to test model fit.

#### Aim 2: Plan of analysis

Using Mplus, we will test the three hypotheses that coalitions receiving the Coalition Check-Up will implement EBPs with greater (1) quantity, (2) quality, and (3) sustainability as compared to the “TA as usual” condition. Prior to hypothesis tests, we will test for baseline differences on each of these dependent variables.

To test aim 2’s first hypothesis, we will model EBP quantity as a coalition-level dependent variable. Once we have three time points of data, we will estimate the developmental trajectory of EBP quantity with growth models—following aim 1 procedures to identify the best functional form of the growth model. Once the developmental trajectory of EBP quantity is properly modeled, it will be predicted by the experimental condition (0=control; 1= Coalition Check-Up) [62].

For the second hypothesis—i.e., programs implemented by coalitions in the Coalition Check-Up condition have superior implementation quality—the unit of analysis is program level, nested within coalition level. Thus, the analytic model will estimate a random intercept. As in the first hypothesis, growth modeling procedures will estimate the developmental trajectory of EBP quality. Experimental condition (0=control; 1= Coalition Check-Up) will be entered at the level of the coalition. Program type (i.e., school-based curriculum, therapist-driven, mentoring, parent curriculum) and program complexity will be covariates at the program level.

For the third hypothesis—i.e., programs implemented by Coalition Check-Up coalitions have superior sustainability—we will test overall EBP sustainability and sustainability planning separately. Coalition Check-Up impact on sustainability planning will be estimated with a multilevel model accounting for the nesting of programs within coalitions. Program-level covariates include program type, program complexity, and baseline EBP longevity. Since overall EBP sustainability is a coalition-level dependent variable, the regression model will not need to adjust for the nesting of programs within coalitions. As with prior models, experimental condition (0=Control; 1= Coalition Check-Up) will be entered at the coalition level.

Mediation analyses will test whether coalition capacity mediates between intervention condition and EBP implementation. We will test whether coalition capacity has significant direct and indirect effects on each EBP implementation measure: (a) quantity, (b) quality, and (c) sustainability. Each of these outcomes will be modeled as previously described in this aim, with the addition of the coalition capacity mediational pathway [63, 64].

#### Aim 3: Analyses

The final aim will be to test the hypothesis that communities receiving the Coalition Check-Up will reduce youth substance use relative to communities in the “TA-as-usual” condition. Youth reports of substance use will be nested in school district (i.e., community or coalition level) via multilevel models. We will start by testing for differences in baseline levels of youth substance use between the two experimental conditions, to examine balance after randomization. If these comparisons identify a difference between the two conditions on a particular measure, it will be included as a covariate in the modeling. Then, youth substance use outcomes in year 3 will be predicted by experimental condition. A longer-term analysis of the Coalition Check-Up’s impact on youth substance use becomes possible with year 5 outcome data. These longer term outcomes will capture the effect of the Coalition Check-Up delivered as a continuous quality improvement cycle over years 2–4 of the study. We will use the same analytic models in year 5 as year 3, given that the data is identifiable at the school-district level but not the student-level.

We will examine 8 youth substance use outcomes (see the “Aim 3: Measures—youth substance use” section) in two multivariate multi-level models [62]. The first model will test past 30-day use of alcohol, tobacco, marijuana, and opioids simultaneously within the coalition’s catchment area [62]. The second model will test lifetime usage outcomes of the same substances. If an overall significant effect is found for the Coalition Check-Up in a particular multivariate model, follow-up tests will examine the specific substances as separate dependent variables [62]. These models extend analyses of Pennsylvania Youth Survey data conducted by Chilenski and Feinberg by taking advantage of increased levels of survey participation, the availability of community-level pretest measures of youth substance use, and the collection of detailed EBP implementation data [24, 59, 65].

Building from models of the Coalition Check-Up’s overall effects on youth substance use, we will test whether EBP implementation mediates between the experimental condition and youth substance use. We will test the EBP implementation measures described in Aim 2 (quantity, quality, sustainability) in separate mediation models. Each model will test for both direct and indirect effects on youth substance use—aggregate 30-day use, and aggregate lifetime use measures of alcohol, tobacco, marijuana, and opioids. If a mediational pathway is significant, we will conduct follow-up analyses modeling each of the specific substances separately [63, 64].

### Cost tracking

Throughout the project, we will assess coalition activity costs to understand the resources necessary for Coalition Check-Up implementation. This assessment will use methodology developed in prior work including annual average cost estimates as well as ranges of costs across differing coalition settings [66]. We will adopt an ingredients-based approach where all program inputs and activities in the day-to-day implementation are identified regardless of whether a direct cost was required [67]. We will seek cost information from multiple sources. Where available, project budgets indicate amounts for personnel and non-personnel inputs. For personnel costs, we will use Coalition Check-Up time allocation measures developed for this project as part of the TA Record [68]. When costs are not apparent (e.g., in budgets), we will derive estimates based on opportunity cost or market value of the resource. Including such valuations enables creation of a range of cost estimates, where the lower bound excludes opportunity costs and the higher bound reflects the value of non-budgeted resources. This process will set the foundation for cost-effectiveness analyses pursued in future projects.

### Dissemination and data sharing

To make results available to both scientists and practitioners, we will pursue presentations at scientific meetings, presentations to community coalitions, brief reports and press releases on the EPIS website (http://epis.psu.edu/), and publication in peer-reviewed journals. Upon request, we will share de-identified data from published studies with interested investigators once they have IRB approval.

## Discussion

This innovative trial tests a unique approach to supporting community coalitions’ implementation of EBPs. Other low-cost TA models are typically expert driven and specific to a single type of coalition [16]. In contrast, the Coalition Check-Up supports coalition-driven action planning. Unlike existing TA models, the Coalition Check-Up can also be applied broadly across coalition models because it focuses on common dimensions of coalition capacity and EBP implementation linked to youth outcomes [20, 21]. The proactive, data-driven monitoring in this study involves continuous quality improvement cycles that identify TA needs and provide timely support to improve capacity [69]. The Coalition Check-Up is unique in its use of motivational interviewing to support its audit and feedback implementation strategy, thereby strengthening the collaborative working relationship, enhancing coalition-driven action planning, and coalition commitment to making change [70, 71].

Using the Interactive Systems Framework and an extensive history of coalition research, this study will examine causal pathways from TA to EBP implementation and youth outcomes [22, 24, 26]. In this way, the study advances the field of implementation science by providing new evidence of the relationship between Interactive Systems Framework domains and implementation outcomes. The scale of this project allows for longitudinal testing of mediating pathways from Coalition Check-Up to distal community-wide outcomes. Together, these innovations will yield evidence about not only if but also how the Coalition Check-Up works. Advancement of the Coalition Check-Up evidence-base will encourage model usage as a low-cost and generalizable approach for supporting coalitions to achieve community-wide reductions in youth substance use.

Each study aim is well-positioned to make important contributions to the implementation science literature. The first aim contributes knowledge of how the Coalition Check-Up affects coalition capacity, by operationalizing the pathway from the prevention support system (TA) to the prevention delivery system (EBP implementation) in the Interactive Systems Framework. These analyses are an important step toward understanding how the Coalition Check-Up might work to support positive outcomes for youth in communities. This knowledge will also help providers and policy makers understand critical components of the prevention support system. Findings will provide guidance on how the prevention support system can best build capacity to address youth substance use in communities across the country.

The second aim will show how the Coalition Check-Up does or does not contribute to aspects of EBP implementation. By examining the Coalition Check-Up as a strategy to support the adoption and integration of EBPs into community settings, we can clarify pathways that improve participant outcomes and benefit population health. Such work is important in not only establishing the evidence for this coalition-driven TA system, but also for understanding the relations between the prevention support system and the prevention delivery system as outlined by the Interactive Systems Framework. Analyses will provide empirical evidence on key implementation processes that support EBPs according to the Interactive Systems Framework.

The third aim will provide insight into how to reduce youth substance use. A granular understanding of how the Coalition Check-Up and EBPs in the prevention delivery system can reduce youth substance use will strengthen evidence bases for both the Interactive Systems Framework and the Coalition Check-Up. By understanding how EBP quantity, quality, and sustainability relate to youth outcomes, policy makers and funding agencies can make better decisions about where to focus scarce EBP dissemination and implementation resources. Furthermore, TA providers will be better able to direct coalitions towards EBP approaches that maximize impact on youth substance use. By reflecting the imperfections of real-world practice, our findings will be more generalizable than in studies where coalitions and EBPs are well funded.

### Limitations

A potential challenge of this project is keeping the coalitions engaged in the Coalition Check-Up and focused on the development and implementation of action plans addressing prioritized areas for growth. Success in this regard may depend on a strong relationship between the TA provider and the coalition, which we include in our fidelity monitoring protocol. TA use of motivational interviewing should help in promoting a strong relationship and in encouraging coalitions to implement their action plans [72].

Another problem could be lower than expected participation in Pennsylvania Youth Survey or the Missouri Student Survey. If we identify school districts in participating communities without student data, a new data collection incentive plan will provide financial incentive for missing school districts to participate in data collection. We will discuss with the state-level survey sponsors to see what logistical support we can offer schools in missing districts.

### Conclusion and impact

Results are expected to have a positive impact on the field by establishing the evidence-base for a low-cost, broadly applicable TA model supporting sustained implementation of evidence-based drug prevention programs and policies.

## Data Availability

Please contact the lead author for more information.

## Abbreviations

EBP: Evidence-based practices
EPIS: Evidence-base Prevention and Intervention Support project
PROSPER: PROmoting School-community-university Partnerships to Enhance Resilience
TA: Technical assistance
